# Hydro-jet propelled colonoscopy: proof of concept in a phantom colon

**DOI:** 10.1007/s00464-020-08089-z

**Published:** 2020-10-22

**Authors:** Stuart A. Coleman, Markus Pakleppa, Alfred Cuschieri

**Affiliations:** 1 Institute of Medical Science and Technology, University of Dundee, Dundee, DD21FD UK; 2 Discipline of Engineering, University of Dundee, Dundee, UK

**Keywords:** Colonoscope, Water, Jet, Propulsion, Colon, Phantom

## Abstract

**Background:**

Colonoscopy is a widely used and effective procedure, but it often causes patient discomfort and its execution requires considerable skill and training. We demonstrate an alternative approach to colonoscope propulsion with the potential to minimise patient discomfort by reducing the forces exerted on the colonic wall and mesentery, and to reduce the level of skill required for execution.

**Methods:**

A prototype colonoscopic device is described, consisting of a tethered capsule that is propelled and manoeuvred through a water-filled colon (hydro-colonoscopy) by an array of water jets. As an initial proof of concept, experiments were performed to assess the ability of the device to navigate through a simplified PVA cryogel human colon phantom arranged in various anatomical configurations.

**Results:**

The prototype was capable of successfully navigating through three out of four colon configurations: a simple layout, alpha loop and reverse alpha loop. It was unable to negotiate the fourth configuration involving an “N loop”, but this was attributed to problems with the colon phantom. In the successful test replicates, mean complete insertion (i.e. caecal intubation) time was 4.7 min. Measured pressures, temperatures and forces exerted on the colon appeared to be within a physiologically acceptable range. The results demonstrate the viability of propelling a colonoscope through a colon phantom using hydro-jets.

**Conclusions:**

Results indicate that this approach has the potential to enable rapid and safe caecal intubation. This suggests that further development towards clinical translation is worthwhile.

Flexible push colonoscopy remains the gold standard test for the diagnosis of colorectal disease. Although it is safe and used widely, it causes patient discomfort, largely due to the colonoscope pressing on the colonic walls as it is pushed through flexures, especially during looping [1]. This discomfort reduces patient compliance for routine colonoscopy [2] and means that the procedure is usually carried out under sedation. Colonoscopy is also a technically difficult procedure, requiring substantial training to acquire the skills required for proficient execution [3]. Furthermore, a substantial and costly amount of physician time is required to advance the colonoscope through the colon prior to detailed colonoscopic examination [4].

These issues may be addressed using an alternative means of colonoscope propulsion, instead of push colonoscopy. Various alternative propulsion approaches have been reported [5–9]; however, none have substantially displaced the conventional colonoscope as a diagnostic tool, probably due to their reduced functionality and increased cost [10]. The most widely used of these alternatives is capsule endoscopy, but currently, this has major limitations as it cannot procure biopsy samples or provide endoscopic therapy.

One possible alternative colonoscope propulsion approach is to utilise jets of pressurised water to propel and manoeuvre a device through the colon. Hydro-jets have been proposed previously for colonoscope propulsion [11] and for manoeuvring a gastroscope [12]. This approach has the potential to reduce patient discomfort compared to conventional colonoscopy as it allows for a highly flexible, self-propelled device that can reduce the forces stretching the colonic walls and mesenteries. Furthermore, during the procedure, the colon is filled with warm water (hydro-colonoscopy), which has been shown to be reduce patient discomfort in conventional colonoscopy [13]. Hydro-jet propulsion also has the potential to reduce user skill requirement as it has a simple control scheme and does not involve the complex manoeuvres that are often required in conventional push colonoscopy, such as torqueing and pulling to straighten loops. This paper is an initial proof of concept of hydro-jet colonoscope propulsion, where the ability of a prototype device to navigate through a synthetic colon model is assessed.

## Materials and methods

### Hydro-jet colonoscope

**Fig. 1:**
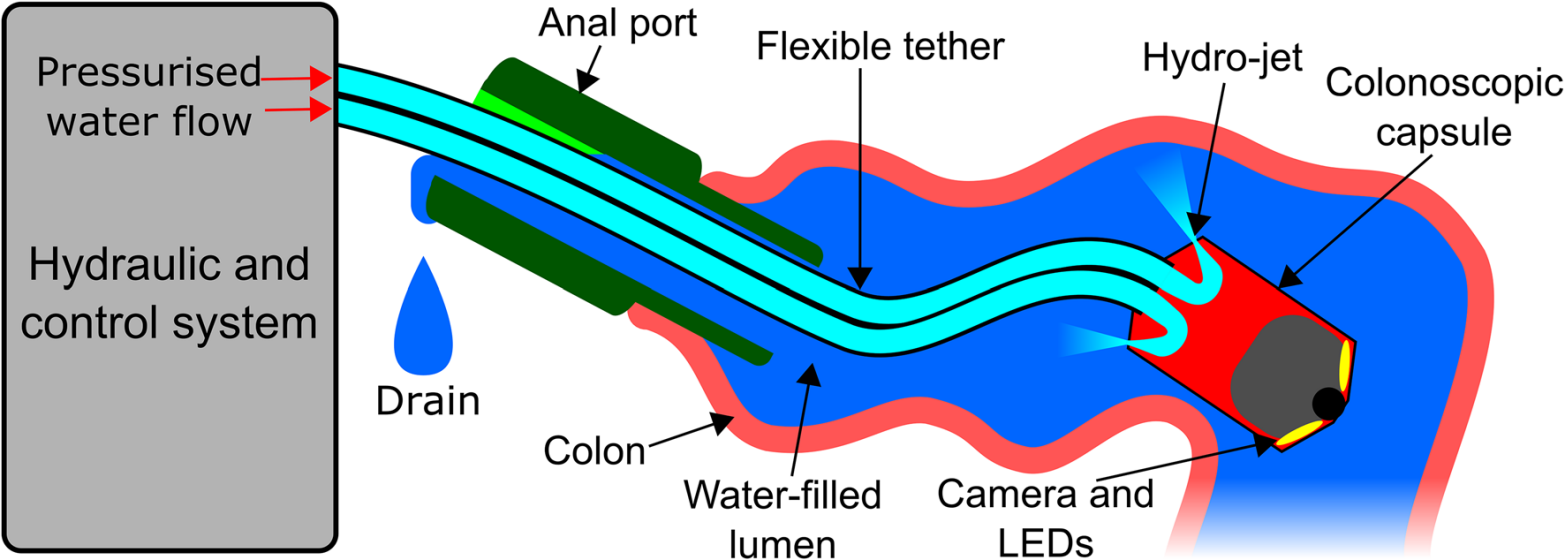
Overview of the HJC

An overview of the hydro-jet colonoscope (HJC) system is shown in Fig. 1. The HJC consists of a colonoscopic capsule which is supplied with pressurised water from an extracorporeal hydraulic system via a flexible tether. This capsule is propelled by water flowing through an array of hydro-jet nozzles; the flow through these nozzles can be selectively controlled to manoeuvre the capsule. Water issuing from the nozzles fills the colon and flows out of an anal port, into an external drain. The HJC is controlled by the user based on feedback from sensors and an on-board camera.

**Fig. 2:**
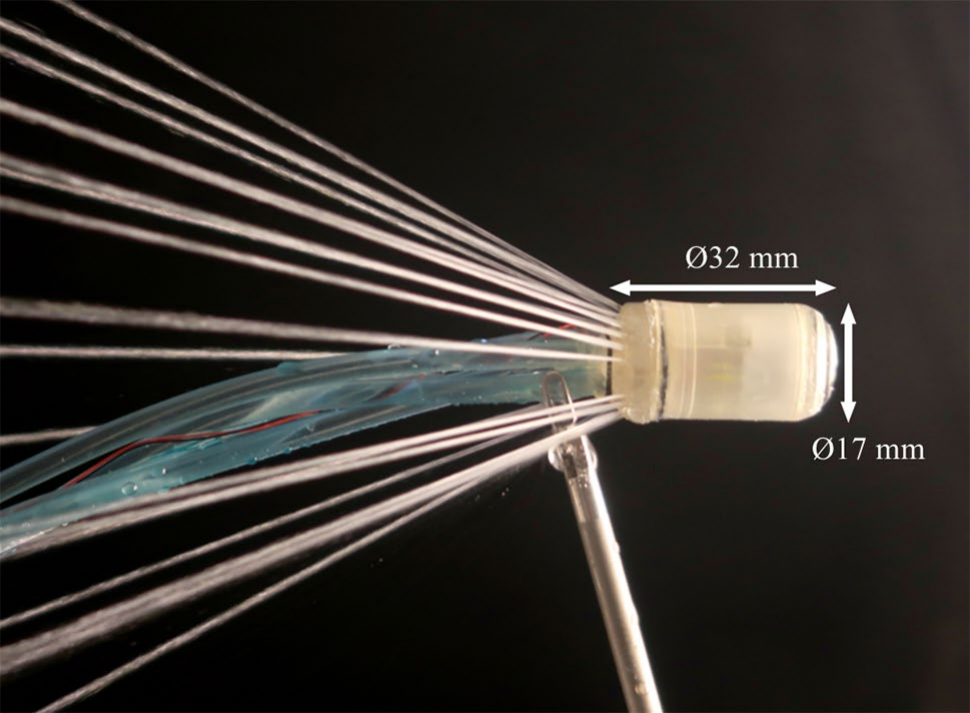
Colonoscopic capsule, with all 20 hydro-jets active. This image shows the jets operating in air; the capsule is typically submerged during operation, in which case the jets will rapidly diffuse in the surrounding water

The colonoscopic capsule is a cylindrical body with twenty Ø 0.5-mm jet nozzles arrayed around its proximal end, as shown in Fig. 2. These are divided into four clusters of five adjacent nozzles; each cluster is supplied with water by an independent flexible tube which forms part of the tether. The capsule can be manoeuvred by supplying each cluster with water at a different pressure. A custom camera and LED illumination module are mounted at the distal end of the capsule. This captures 1080p, 30 frames per second video with a field of view of approximately 95° in water. The capsule is Ø 17 mm × 32 mm, has a mass of 7 g and is designed to be neutrally buoyant in water.

The capsule is connected to an extracorporeal hydraulic system by a 1.9-m-long tether, which is about 100 times more flexible than a conventional colonoscope [14]. The hydraulic system consists of a pump, valves and sensors. It supplies water at a pressure of up to 370 kPa, with flowrates of up to 75 ml/s and at a temperature of approximately 37 °C. These parameters were selected based on preliminary studies, including experimental studies of hydro-jet impingement on excised porcine colon samples, which indicate that the hydro-jets are unlikely to cause tissue damage and should generate adequate thrust for propulsion. The pressure, flowrate and temperature of the water supplied from the hydraulic system are monitored, and the pump is automatically shut off if these exceed pre-defined safe limits. Furthermore, if the water supply or electrical power is interrupted, the hydraulic system will passively cut out, and the HJC can be withdrawn manually.

At the start of the procedure, the capsule is introduced into the colon along with the anal port. The capsule is initially mounted at the front of the anal port for visual guidance of its insertion. The anal port is connected to an elevated drain, enabling maintenance of a hydrostatic intraluminal water pressure of approximately 2 kPa (15 mmHg) during use. The height of the drain, and hence the intraluminal pressure, can be adjusted to ensure adequate distension of the colonic lumen. The anal port also contains an intraluminal pressure sensor and a roller to reduce friction where the tether slides through the port.

The HJC is controlled by the user based on feedback from the on-board video camera, pressure sensors and flowrate sensors. The user may control the HJC in three ways: firstly, using a foot pedal to vary the pressure of the hydro-jets, hence adjusting the thrust generated like a “throttle”; secondly, using a joystick to turn the capsule by selectively modulating the flow to the clusters of nozzles and thirdly, the user may directly interact with the tether, i.e. pushing, pulling or holding it to prevent or encourage movement of the capsule and tether. Due to its high flexibility, the tether will buckle when it is pushed; hence, it is impossible to push the capsule through the colon.

Control and user feedback are provided via a PC running control software (LabVIEW, National Instruments Corp., USA). The assembled hydraulic and control systems are contained in a small trolley (1 m × 0.6 m × 0.4 m), which is located next to the patient undergoing colonoscopy. Further technical details of the HJC design are pending publication [15].

### Test environment

An artificial test environment was developed to represent the human colon for testing the insertion of the HJC. This environment consisted of a custom colon phantom mounted in the rigid base of a colonoscopy simulator representing the abdominal cavity.

The colon phantom was moulded from polyvinyl alcohol cryogel (PVA-C) in a simple tubular shape, with an inside diameter of 44 mm and length of 1.5 m. The elastic modulus of this material was tested and found to be lower than mean reported values for the colon [16], so the wall thickness of the tube was increased to compensate, resulting in a tube of similar wall stiffness. Previous work [17] shows that PVA-C with a similar formulation and production methodology has a coefficient of static friction of approximately 0.03 when tested against smooth, hard material. This is broadly consistent with the values reported for the colon: 0.03–0.25 [17, 18].

The colon phantom is mounted in a rigid enclosure shaped to represent the human abdominal cavity. This enclosure is part of a commercially available colonoscopy training model (Kyoto Kagaku Co. Ltd., Japan). The colon phantom is constrained within the enclosure using attachments provided with the training model—these are flexible rings mounted to the enclosure either directly or via a flexible coiled cord. The number and location of these attachments may be varied to simulate different anatomical configurations of the colon. Experiments were carried out with four different colon configurations, which correspond to training scenarios described in the manual supplied with the colonoscopy training model [19]:

A. A colon configuration resembling the simple “textbook” colon. This is a relatively short and direct configuration, with minimal redundancy. The ascending and descending colons are well constrained, whereas the sigmoid and transverse colons are relatively mobile. This scenario is “introductory level II” in the training manual.
B. Variation of configuration “A” with an elongated sigmoid colon shaped into a pre-formed alpha loop. This scenario is “primary level” in the training manual.
C. Variation of configuration “A” with a highly elongated, redundant sigmoid colon shaped into an “N” loop configuration. The transverse colon is also elongated and poorly constrained. This scenario is “secondary level” in the training manual.
D. Variation of configuration “A” with an elongated sigmoid colon shaped into a reverse alpha loop configuration (i.e. the colon loops back under itself). This is the final “advanced level” scenario in the training manual.

**Fig. 3:**
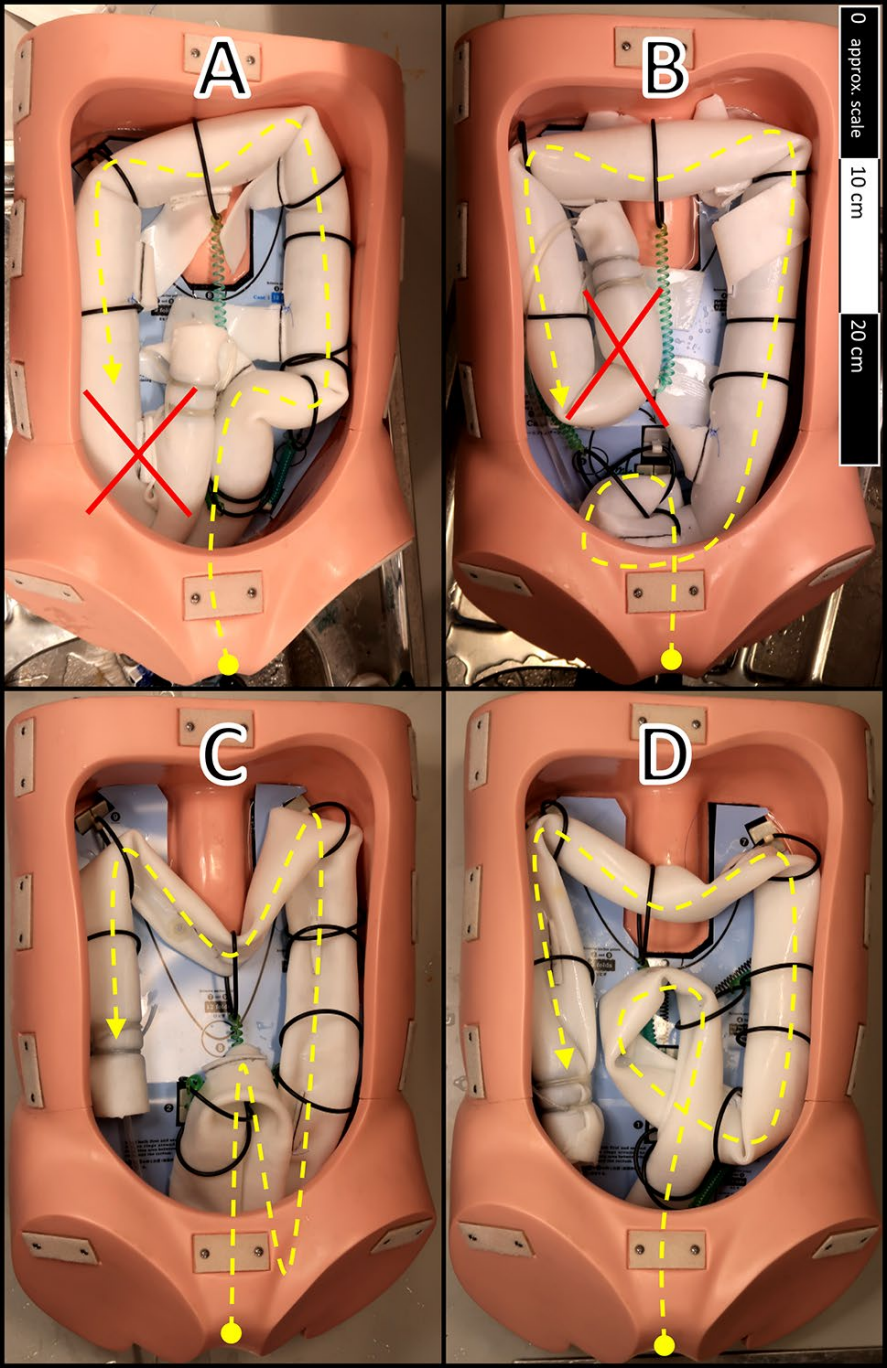
Colon configurations **A**–**D**. These configurations are based on [19] and correspond to variations in colonic anatomy. In all configurations, the colon phantom is arranged in an abdominal enclosure and secured at various attachment points. In configurations **A** and **B**, a portion of the colon phantom is not required in the layout—this portion is marked with a red cross. In these cases, a marker is placed at the designated endpoint to show completion (Color figure online)

These configurations are illustrated in Fig. 3. In all configurations, the test environment is fixed in a supine position.

## Procedure

Prior to each test, the HJC is run for 1 min to warm the water in the system and the anal port is inserted into the test environment. The test starts with the HJC being used to fill the colon with water. This is achieved by pumping water through the HJC, while the capsule is retained in the anal port. After filling is complete, the user releases the capsule and propels it through the colon, with the goal of reaching the caecum. The user may adjust the height of the drain to alter the intraluminal pressure if required. The test ends when the capsule reaches the caecum or is unable to progress further through the colon.

For each colon configuration (A–D), four replicate runs of the procedure were carried out. For each test replicate, the pressure, flowrate and temperature of the supplied water, the test duration, and the intraluminal pressure at the anus and caecum were recorded. Pressures were measured relative to the base of the anus which is the lowest point of the lumen and hence has the highest hydrostatic pressure. After testing, mean results and standard deviation were calculated for each colon configuration.

All tests were carried out by the same individual who had not previously performed a colonoscopy. Prior to testing, the individual was given one hour to practice with the HJC in the test environment, using a colon configuration different from the experimental configurations. As testing was not carried out on a human subject, IRB approval was not required.

## Results

**Fig. 4:**
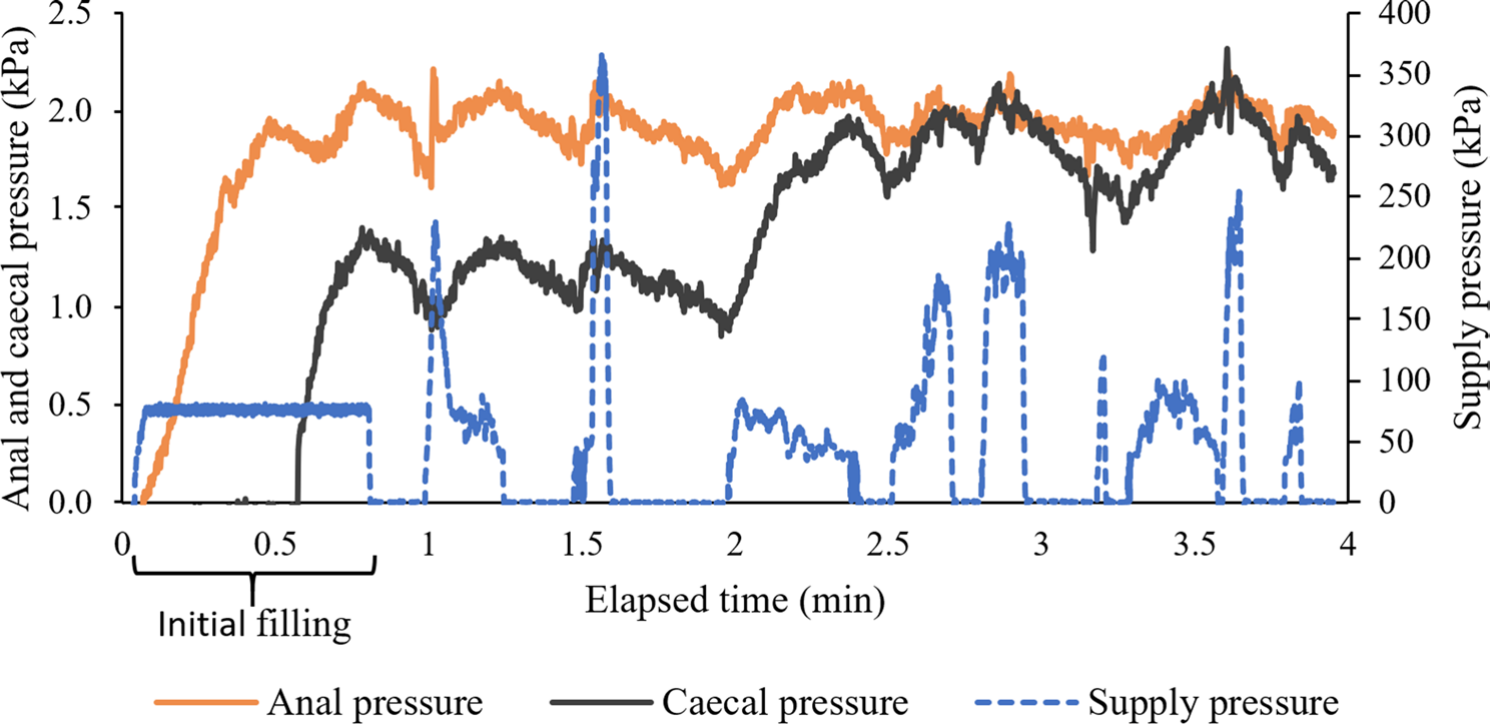
Variation in pressure during one replicate run of configuration D. A common feature from all test replicates is an initial filling period, characterised by constant supply pressure. Supply pressure was generally increased by the user while attempting to navigate flexures; any relatively straight sections of colon were traversed rapidly and easily

The caecum was successfully reached in all replicates for colon configurations A, B and D. In all replicates for configuration C, the capsule could only be advanced partially through the sigmoid colon. In two of these replicates, the capsule could not be manoeuvred past the first flexure, while in two other replicates, it was possible to proceed to the second sigmoid flexure. The results are summarised in Table 1. Figure 4 shows an example of the measured pressures during one test run.

**Table 1:** Summary of test results for the four colon configurations (mean ± standard deviation)

Colon configuration	A	B	C	D
Success rate (%)	100	100	0	100
Insertion time (min)	3.7 ± 0.9	6.4 ± 2.1	5.1 ± 1.7	4.0 ± 0.2
Mean supply pressure (kPa)^a^	149 ± 22	188 ± 19	273 ± 35	157 ± 41
Max supply pressure (kPa)	331 ± 44	397 ± 20	413 ± 63	396 ± 22
Mean supply flowrate (ml/s)^a^	40 ± 6	49 ± 3	63 ± 5	42 ± 8
Max. supply flowrate (ml/s)	74 ± 5	77 ± 1	81 ± 0	76 ± 8
Mean anal pressure (kPa)	2.2 ± 0.3	1.8 ± 0.3	2.6 ± 0.4	1.8 ± 0.1
Mean caecal pressure (kPa)	2.2 ± 0.2	1.8 ± 0.2	2.0 ± 0.9	1.2 ± 0.2
Max. intraluminal pressure (kPa)	3.2 ± 1.1	2.8 ± 0.2	3.9 ± 0.3	2.7 ± 0.3
Water volume supplied (l)	5.3 ± 1.2	11.7 ± 4.1	12.6 ± 2.9	5.8 ± 0.9

^a^Averaged over times when hydro-jets are active, after the initial filling of the colon

## Discussion

### Performance

**Fig. 5:**
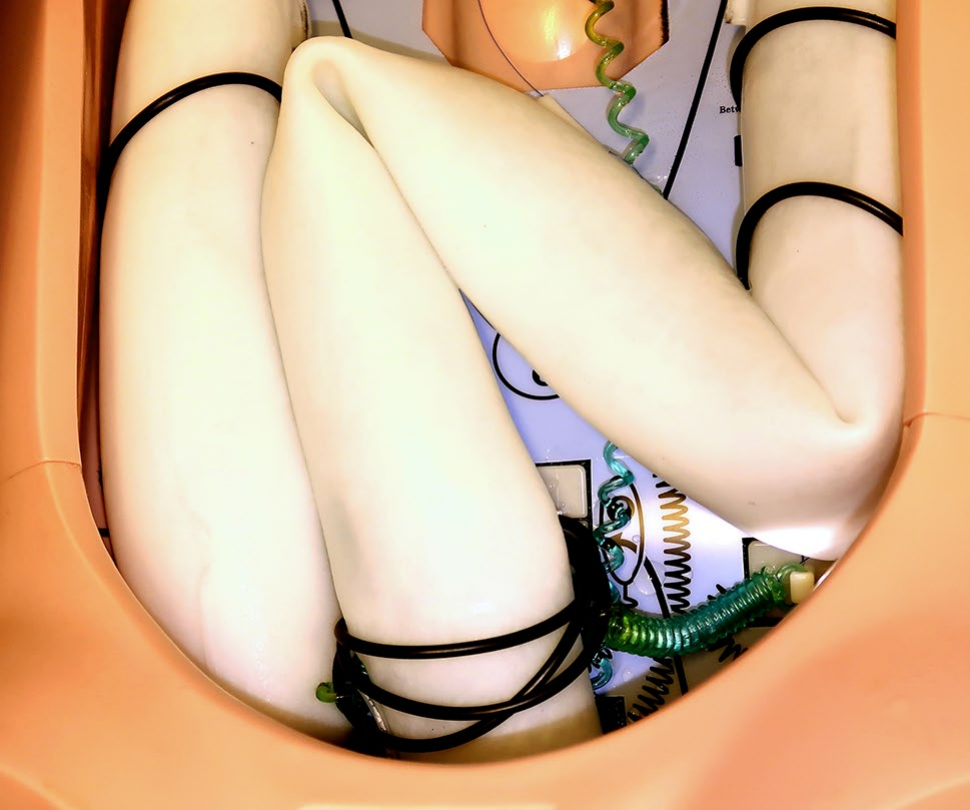
"N" loop in configuration C, with two acute buckled flexures

Successful insertion of the HJC to the caecum in colon configurations A, B and D demonstrates that the HJC is capable of navigating through various challenging colon configurations, including loops and multiple tight flexures. However, the HJC was incapable of negotiating the sigmoid colon in configuration C. This consisted of a loosely constrained, redundant “N” loop exhibiting two acute flexures with tight radii, as shown in Fig. 5. At each of these flexures, the lumen of the colonic phantom was almost entirely occluded due to buckling. This occurred in every test replicate and persisted even when intraluminal pressure was increased to 4 kPa. As this type of buckled occlusion is not reported in the clinical literature, it represents, in our view, an unrealistic difficulty.

The maximum instantaneous intraluminal pressure measured during testing was 5.2 kPa. This corresponded to hydro-jets impinging near the pressure sensor. Excluding this event, the highest observed pressure was 4.2 kPa, which is well within the range of intraluminal pressure used in clinical colonoscopy [20]. A preliminary study of water jets impinging on excised porcine colon samples suggests that the jets in the HJC prototype should be safe for use in the colon. The mean volume of water used in the tests was 8.9 L. Approximately 2 L of this was required to fill the colon, while the remainder flowed through the colon and out of the drain. This water flow could cause hyperthermia or hypothermia, known to occur during retrograde colonic lavage [21]. However, as the mean water supply temperature during the experiments (35.9 °C) approximates body temperature, heat transfer should be minimal and is unlikely to be hazardous. The fluid used in the hydraulic system was tap water; however, for clinical use, it would be preferable to use an isotonic liquid to minimise fluid absorption and electrolyte shifts [22].

The HJC was not experimentally compared to conventional colonoscopy. However, a study using a similar test environment and colon configurations reported mean colonoscope insertion times for experienced colonoscopists of between approximately 2 min (for configuration A) and 10 min (for configuration C) [23]. The test environment and methodology in this study are not identical to ours so cannot be directly compared, but these results suggest that the HJC insertion time (mean: 4.7 min in successful replicates) is broadly comparable to conventional colonoscopy.

The study [23] also reported peak forces applied to the test environment during colonoscopy. These ranged from 11 to 23 N for experienced colonoscopists and 15 to 24 N for novices, values which are broadly consistent with colonoscopy forces applied in vivo [24]. In contrast, the maximum available hydro-jet thrust was measured as 1.2 N, and the maximum compressive load supported by the tether before buckling was approximately 0.3 N. Therefore, the maximum load applied by the HJC is ≤ 1.5 N. This reduction in force is possible because the HJC is self-propelled and uses a highly flexible tether. Hence, it can be concluded that the HJC reduces contact forces on the colon compared to conventional colonoscopy, which should reduce patient discomfort.

The HJC’s simple controls and direct advancement without the need for complex manoeuvres appeared to be intuitive for an inexperienced user to use. Despite this, precisely advancing the capsule down the centre of the lumen was challenging, with the capsule frequently impacting the colonic wall. Greater precision of control would be required for targeted procedures such as biopsy.

In some test replicates, gas pockets were encountered due to residual air in the colonic phantom. These were not an impediment to progress as the capsule could be propelled through them. However, the interaction of the hydro-jets and gas pockets would often fill the surrounding water with bubbles, reducing visibility. At times, it was necessary to pause advancement and wait for these bubbles to clear.

### Test environment

To maximise the validity of the test environment for studying colonoscope insertion, characteristics affecting its mechanical interaction with a colonoscope were prioritised during development. The colon phantom has a coefficient of friction consistent with values observed for the human colon and a similar wall stiffness. The constraints and layouts used are adopted from a colonoscopy trainer with proven construct validity [23]. Compromises were made in that the colonic phantom has a simplified tubular geometry, lacking haustra and variation in diameter.

By developing a novel PVA-C colon phantom, it was possible to mimic the frictional properties of the colon without relying on a lubricant that could be washed or wiped away during testing. The PVA-C phantom was able to withstand repeated testing without observed wear or degradation and was thus easier to work with than biological tissue; however, PVA-C must be stored in water when not in use to prevent dehydration. The colon phantom exhibited a problematic tendency to buckle, although this can likely be addressed in future by modifying the geometry and material formulation.

### Limitations

The study was designed as an initial assessment of the ability of an HJC to navigate through a colon, because this is the greatest area of uncertainty regarding the viability of HJCs. The experiments were carried out in a synthetic colon phantom, which mimicked key characteristics of the human colon but had some discrepancies from in vivo anatomy. The measurements obtained during testing are indicative of successful insertion rate, patient comfort and safety; however, confirmation of these results by in *vivo* experiments is required. The study only involved a single user, so further testing would be required to assess the user skill requirement of the HJC.

The experiment was focused on colonoscope insertion (caecal intubation) as this is typically the most painful and technically challenging stage of the procedure. However, colonoscope withdrawal (extubation) is the most important stage for clinical assessment. Further experiments are therefore required to assess this part of the procedure, including the HJC’s ability to detect legions accurately and in a timely manner. The ability to carry out targeted tasks such as biopsy and polypectomy is also an important functionality that requires assessment and possibly further development, initially in an ex vivo environment.

## Conclusions

A prototype colonoscopic device is presented consisting of a tethered capsule that can be propelled and manoeuvred using an array of hydro-jets. This device was tested in a novel PVA-C colon phantom constrained within an abdominal enclosure. Testing was carried out in a range of colon configurations, including loops, tight flexures and redundant colons. These configurations are established as challenging in clinical colonoscopy and were thus a test of the HJC’s ability to negotiate taxing colonic configurations.

When controlled by an inexperienced user, the HJC was able to successfully negotiate three of the four tested colon configurations, demonstrating its ability to traverse a long and convoluted colon. In the remaining configuration, the HJC was unable to pass through a series of flexures where the colonic lumen had buckled closed as an artefact of the colon phantom used. Possible improvements to the HJC and test environment were identified.

Caecal intubation times were broadly comparable to those achieved with conventional colonoscopy in similar scenarios. Pressure, temperature and flowrate data indicate that damage to the colon was unlikely. As the HJC is unable to exert large forces on the colon, it has the potential to reduce patient discomfort compared to a conventional colonoscopy. Overall, the results indicate that that further development of this approach towards clinical translation is worthwhile.
